# Altered Gut Microbial Metabolites in Amnestic Mild Cognitive Impairment and Alzheimer’s Disease: Signals in Host–Microbe Interplay

**DOI:** 10.3390/nu13010228

**Published:** 2021-01-14

**Authors:** Li Wu, Yuqiu Han, Zhipeng Zheng, Guoping Peng, Ping Liu, Siqing Yue, Shuai Zhu, Jun Chen, Hanying Lv, Lifang Shao, Yan Sheng, Yulan Wang, Liang Li, Lanjuan Li, Baohong Wang

**Affiliations:** 1 State Key Laboratory for Diagnosis and Treatment of Infectious Diseases, National Clinical Research Center for Infectious Diseases, Collaborative Innovation Center for Diagnosis and Treatment of Infectious Diseases, The First Affiliated Hospital, Zhejiang University School of Medicine, Hangzhou 310003, China; cfightive@zju.edu.cn (L.W.); 11918221@zju.edu.cn (Y.H.); zpzheng@zju.edu.cn (Z.Z.); 11818164@zju.edu.cn (S.Z.); 21918242@zju.edu.cn (J.C.); 15168236539@163.com (H.L.); 1192038@zju.edu.cn (L.S.); ljli@zju.edu.cn (L.L.); 2 Department of Neurology, The First Affiliated Hospital, Zhejiang University School of Medicine, Hangzhou 310003, China; pgpfc@163.com (G.P.); liuping908@163.com (P.L.); 3 Key Laboratory of Microbial Technology for Industrial Pollution Control of Zhejiang Province, College of Environment, Research Center of Environmental Science, Zhejiang University of Technology, Hangzhou 310032, China; yuesiqing@126.com; 4 Department of Ophthalmology, The First Affiliated Hospital, Zhejiang University School of Medicine, Hangzhou 310003, China; yansheng326@126.com; 5 Singapore Phenome Center, Lee Kong Chian School of Medicine, Nanyang Technological University, Singapore 308232, Singapore; yulan.wang@ntu.edu.sg; 6 Department of Chemistry, Alberta University, Edmondon, AB T6G 2G2, Canada; Liang.Li@ualberta.ca; 7 Research Units of Infectious disease and Microecology, Chinese Academy of Medical Sciences, Hangzhou 310003, China

**Keywords:** Alzheimer’s disease, fecal metabolomics, intestinal microbiota, tryptophan, short-chain fatty acids

## Abstract

Intimate metabolic host–microbiome crosstalk regulates immune, metabolic, and neuronal response in health and disease, yet remains untapped for biomarkers or intervention for disease. Our recent study identified an altered microbiome in patients with pre-onset amnestic mild cognitive impairment (aMCI) and dementia Alzheimer’s disease (AD). Thus, we aimed to characterize the gut microbial metabolites among AD, aMCI, and healthy controls (HC). Here, a cohort of 77 individuals (22 aMCI, 27 AD, and 28 HC) was recruited. With the use of liquid-chromatography/gas chromatography mass spectrometry metabolomics profiling, we identified significant differences between AD and HC for tryptophan metabolites, short-chain fatty acids (SCFAs), and lithocholic acid, the majority of which correlated with altered microbiota and cognitive impairment. Notably, tryptophan disorders presented in aMCI and SCFAs decreased progressively from aMCI to AD. Importantly, indole-3-pyruvic acid, a metabolite from tryptophan, was identified as a signature for discrimination and prediction of AD, and five SCFAs for pre-onset and progression of AD. This study showed fecal-based gut microbial signatures were associated with the presence and progression of AD, providing a potential target for microbiota or dietary intervention in AD prevention and support for the host–microbe crosstalk signals in AD pathophysiology.

## 1. Introduction

Alzheimer’s disease (AD) is one of the leading causes of disability or death in the elderly worldwide and has become a serious health problem [1]. According to the World Alzheimer Report, more than 50 million people are living with dementia mainly caused by AD and the number of these patients will be 152 million in 2050 [2]. AD typically progresses in three stages: preclinical, mild cognitive impairment (MCI), and dementia [3]. Early detection of AD, especially from amnestic MCI (aMCI) patients with great risk for AD developing [4] and decades of the pathophysiological process of AD, is important for improving prognosis and early intervention of AD. Through decades of research, the prevailing view of AD pathogenesis are deposition of Aβ plaques and vascular pathology [5,6]. However, the pathogenesis of AD remains unclear and no efficient medical treatment is available for AD [3]. To improve the outcomes of AD, studies to understand the perturbation in other pathophysiology pathways of AD, and to detect AD from high-risk populations such as those with aMCI are urgently needed. 

The intestinal microbiota (IM) is considered as an ‘organ’, producing numerous metabolites that interact with host physiology and affect both the local intestine and distant brain functions [7,8]. In humans, several studies have reported the difference of IM in both American [9] and Chinese patients with AD [10]. In germ-free mice, AD exhibited obvious reduction of cerebral amyloid beta, highly indicating a role of IM in early pathological change of AD [11]. Therefore, examination of perturbation in microbial metabolites could help to reveal the microbial signatures that help in improving the prognosis or ameliorating the progression of AD. A recent study reported that metabolomics for detecting fecal metabolites could explore the links between microbiome composition, host phenotypes, and heritable complex traits [12]. The recent metabolomics studies in AD were well conducted in blood [13], urine [14], brain [15], or cerebrospinal fluid (CSF) [16], however, no functional readout of fecal microbiota-metabolomics has been reported. 

In the current study, high-resolution untargeted and targeted fecal metabolomics were performed to profile and quantify the microbial signatures in a Chinese cohort including AD (pre-onset aMCI and early stage AD) patients and a normal cognition healthy control (HC). Furthermore, we related the differential fecal metabolites of AD to intestinal dysbiosis and cognitive impairment. This approach will enable the discovery of potential microbial biomarkers that are associated with AD, which can be utilized for improving early detection of AD, and facilitate a better understanding of the pathogenesis of AD and open up therapeutic opportunities.

## 2. Materials and Methods

### 2.1. Study Subjects

The study was conducted in accordance with the Declaration of Helsinki, and the protocol was approved by the Ethics Committee of the First Affiliated Hospital, College of Medicine, Zhejiang University (2019-251). As described previously [10], the volunteers were recruited consecutively and written informed consent was obtained. All subjects underwent a complete medical history evaluation, physical examination, neurological, and neuropsychological assessment (Mini-Mental State Examination [MMSE] and the Beijing version of the Montreal Cognitive Assessment [MoCA]), neuroimaging (magnetic resonance imaging), and clinical biochemistry examinations. AD was diagnosed according to the criteria of Diagnostic and Statistical Manual (DSM)-IV [17] and guidelines of the National Institute of Neurological and Communicative Disorders and the Stroke and Alzheimer Disease and Related Disorders Association (NINCDS-ADRDA) [18]. The aMCI was diagnosed as described previously [19]. The severity of aging dementia was evaluated by the Clinical Dementia Rating (CDR) [10] with a rating scale ranging from no dementia, to questionable dementia (MCI, CDR 0.5) and ultimately varying stages of dementia (AD, CDR ≥ 1) [19]. The gender- and age-matched normal cognition elders were recruited as healthy controls (HC), and the majority of them were the patients’ spouses, who had lived together and had been on the same diet for at least twenty years.

A cohort of 77 participants was recruited (22 aMCI, 27 AD, 28 HC). Morning fasting serum and fecal samples were freshly collected in the baseline visit [20,21]. The demographic information and clinical characteristics of the cohorts are summarized in Table 1 and Table 2. The sequence dataset of the fecal microbiota can be downloaded from the National Center for Biotechnology Information (NCBI) Bioproject database (PRJNA496408). 

### 2.2. Fecal Metabolomics Analysis

#### 2.2.1. Sample Preparation

The preprocessed pipeline of fecal samples was established. First, the samples were mixed with methanol (3 mL/g) and homogenized (8 m/s, 15 s) with ceramic beads (1 mm) (Omni International, Bedford, NH, USA) [22]. Then, the supernatants after centrifugation were filtered using syringe filters (0.22 μm, Millipore Corp, Billerica, MA, USA). To monitor potential contamination of sample processing, a procedural blank sample of water (Thermo Fisher Scientific, Waltham, MA, USA) was used. Quality control (QC) samples were prepared by mixing equal volumes of all fecal extraction, and processed with the real samples simultaneously. 

#### 2.2.2. Fecal Untargeted Metabolomics Profiling

The metabolomics profiling was performed using a Dionex UltiMate 3000 RS ultraperformance liquid chromatography (UPLC) system coupled with Orbitrap Q Exactive mass spectrometry (MS) with a Hypersil Gold C-18 column (2.1 × 100 mm, 1.9 µm) (Thermo Fisher Scientific, Waltham, MA, USA). Both ESI+ and ESI– ionization modes were executed with a *m*/*z* range 70–1050 and a full MS scan at 60,000 resolution. 

#### 2.2.3. Multivariate Data Analysis

Raw data were acquired by Xcalibur 4.1 software and processed by Compound Discoverer 3.1 software (Thermo Fisher Scientific, Waltham, MA, USA) for peak detection, peak alignment, and peak integration. The processing procedures were modified from a previous method [23]. The multivariate statistical analysis including principle component analysis (PCA) and partial least-squares-latent structure discriminate analysis (PLS-DA) were performed in SIMCA-P 13.0 (Umetrics AB, Umea, Vasterbotten, Sweden) [24]. 

The biologically metabolites were picked out using Compound Discoverer 3.1. Briefly, the local library and online database including ChemSpider and mzCloud, of which the Human Metabolome Database (HMDB), Kyoto Encyclopedia of Genes and Genomes (KEGG), and Biocyc were used. The identity of the candidate biomarkers were confirmed by the standard compound libraries of both our lab and the public database. 

### 2.3. Targeted Profiling of Fecal Tryptophan Metabolites

The mass list of endogenous tryptophan metabolites (Table S1) was compiled using the “Search Mass Lists” node of the Compound Discoverer 3.1, conducting the full-MS scan to pick out the tryptophan metabolites from samples.

### 2.4. Targeted Profiling of Fecal Short-Chain Fatty Acids (SCFAs)

Short-chain fatty acid (SCFA) profiles and quantification were performed by gas chromatography (GC)-MS based on an Agilent 7890B with a single quadrupole MS (5977, Agilent Technologies, Santa Clara, CA, USA). The samples were prepared by mixing with 10% isobutanol, and homogenized twice (50 HZ, 30 s) using tissuelyser (QIAGEN, Hilden, Germany) and centrifuged (12,000 rpm, 5 min). Then, the chloroform derivatization process was modified based on that previously described [25,26]. The identity of SCFAs were based on the retention time and fragments compared with the chemical standards (Table S2) and quantified by external standard calibration methods using Mass Hunter software (Agilent Technologies, Santa Clara, CA, USA) [27].

### 2.5. Targeted Profiling of Fecal Bile Acids

The bile acids (BAs) were determined using an UPLC tandem MS system (6470, Agilent Technologies, Santa Clara, CA, USA). As previously described [28,29], the samples were extracted by methanol solvent containing an internal standard (Table S3), processed with rapid freeze−thaw cycles, centrifuged, and finally filtered (0.22 µm filter). The raw data were processed using Mass Hunter software. The BAs were identified by referring to the retention time and ion pairs of standards (Tables S4 and S5) and quantified by the internal standard calibration curves. 

### 2.6. Measurement of Circulating Lipopolysaccharide Level

The serum lipopolysaccharide (LPS) levels were measured using the limulus amoebocyte lysate (LAL) chromogenic endpoint assay (Hycult Biotech, Uden, The Netherlands) [30] by the spectrophotometer (Biotek, Winooski, VT, USA) with the absorbance at 405 nm.

### 2.7. Statistical Analysis

The univariate analysis of each metabolite was performed by one-way ANOVA or Kruskal–Wallis test using SPSS software package (version 16.0, SPSS, Inc., Chicago, IL, USA) and GraphPad Prism 6 (GraphPad software, La Jolla, CA, USA). A *p* value < 0.05 was considered statistically significance. Multiple comparison corrections were conducted using False Discovery Rate (FDR) correction for adjusted *p* value (or q value). Correlation analysis was performed using Spearman rank correlation analysis. A hierarchical clustering heat map was established by correlation coefficient (Cluster 3.0 software) [31]. The binary logistic regression models were built for biomarkers based on the backward-selected metabolites. The area under the receiver operating characteristic (ROC) curves (AUC) were used to assess the predictive ability of models [10,32]. The schematic workflow of statistical analysis is listed in Figure S1. 

The detailed information of all methods and materials are provided in the Supplementary Materials. 

## 3. Results

### 3.1. Distinguishing Metabolomics Profiles among AD, aMCI, and HC

**Figure 1:**
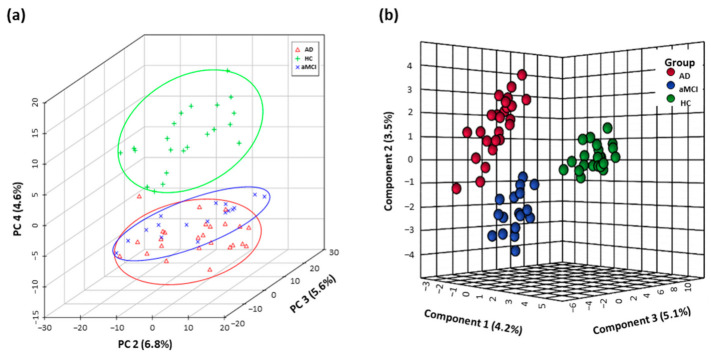
Fecal metabolomics profiles separated AD patients from HC. Differential fecal metabolomics profiles among aMCI (n = 17), AD (n = 25) and HC (n = 22) were presented by (**a**) PCA score plot based on untargeted high-throughput UHPLC-orbitrap Q Exactive-MS metabolic profiling and (**b**) PLS-DA model based on top twenty-five differential metabolites. Abbreviations: HC, normal cognition healthy control; aMCI, amnestic mild cognitive impairment; AD, Alzheimer’s disease; PCA, principle component analysis; PLS-DA, partial least-squares-latent structure discriminate analysis; UHPLC, ultra-high performance liquid chromatography; MS, mass spectrometry.

The fecal metabolomics profiles of patients with pre-onset aMCI and AD were clearly separated from HC individuals in the PCA score plot, and even showed the separation trend between aMCI and AD groups (Figure 1a). Furthermore, based on the top twenty-five differential metabolites, the PLS-DA plot presented the separation among the AD, aMCI, and HC groups (Figure 1b). Interestingly, the altered metabolites in AD mainly pointed to the disturbance of tryptophan metabolism (Table S6).

### 3.2. Disturbed Metabolic Pathways of Tryptophan in Patients with AD

**Figure 2:**
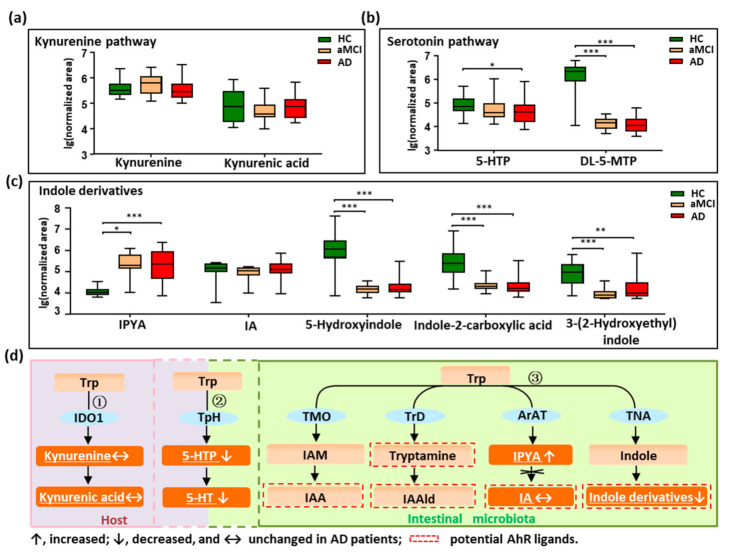
Dysregulation of fecal tryptophan metabolic pathway in AD patients. (**a**–**c**) Comparison of detected tryptophan metabolites in the kynurenine pathway, serotonin pathway, and indole derivatives among AD, aMCI, and HC groups. (**d**) There are three major tryptophan metabolism pathways: ① Kynurenine pathway mainly occurs in the liver or extrahepatic organs; ② Although neurotransmitter serotonin (5-hydroxytryptamine [5-HT]) can be synthesized in the brain, 90% of the body’s 5-HT is produced in the gut; ③ Tryptophan was directly transformed into indole and its derivatives in the gut controlled by gut microbiota, and many of the indole derivatives are potential AhR ligands. The detected tryptophan metabolites in the current study were marked with a white color and underlined. Note: Data are given as median with range. *p* values were determined by one-way ANOVA or Kruskal–Wallis test. * *p* < 0.05; ** *p* < 0.01; *** *p* < 0.001. Abbreviations: Trp, tryptophan; IDO1, indoleamine 2,3-dioxygenase 1; TpH, tryptophan hydroxylase; TMO, tryptophan 2-monooxygenase; TrD, tryptophan decarboxylase; ArAT, aromatic amino acid aminotransferases; TNA, tryptophanase; 5-HTP, 5-hydroxytryptophan; DL-5-MTP, DL-5-methoxytryptophan; IAM, indole-3-acetamide; IAA, indole acetic acid; IAAld, indole-3-acetaldehyde; IPYA, indole-3-pyruvic acid; IA, indoleacrylic acid; AhR, aryl hydrocarbon receptor.

We profiled all metabolites of the tryptophan pathways by setting an in-house mass list library based on KEGG data. As shown in Figure 2a–d, in total, nine tryptophan metabolites were detected and quantified. Metabolites were annotated to the three reported pathways, two in the host (the kynurenine pathway and serotonin pathway), and one in the intestine (indole derivatives pathway). We did not find changes in the kynurenine and kynurenic acid in patients with AD (Figure 2a,d), indicating that tryptophan metabolized by the host was not altered in AD. Interestingly, the findings showed that the metabolites in the serotonin pathway significantly decreased in AD (Figure 2b,d). For example, 5-hydroxytryptophan (5-HTP), a precursor of 5-HT, was significantly decreased in AD compared with HC (*p* = 0.022), and showed a reduced trend in aMCI when compared with HC. Additionally, the 5-HT family member, DL-5-methoxytryptophan (DL-5-MTP), was markedly depleted in both aMCI and AD compared with HC (*p* < 0.001). These findings indicate that the tryptophan co-metabolized by host and microbiota was significantly disturbed in AD. 

Furthermore, we revealed that indole derivatives of the tryptophan pathway metabolized by IM were markedly disturbed (Figure 2c,d). For instance, the derivative indole-3-pyruvic acid (IPYA) was progressively enriched from aMCI and AD compared with HC (*p* < 0.05). Of note, the indole derivatives, acting as the aryl hydrocarbon receptor (AhR) ligands, were markedly depleted in both aMCI and AD when compared with HC (*p* < 0.01). These findings show that the tryptophan pathways metabolized by IM were significantly disturbed in AD, especially the AhR ligands of indole derivatives. After adjusting the significance level for multiple comparisons, these tryptophan metabolites in aMCI and AD patients were still significantly different from HC (*p* < 0.001) (Table S6).

### 3.3. Fecal SCFAs Associated with Progression from HC to aMCI and AD

**Figure 3:**
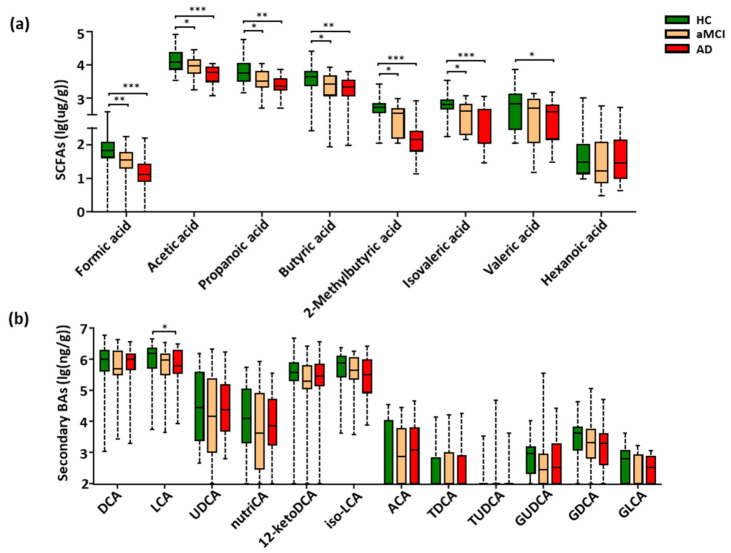
Progressively decreased SCFAs and LCA from HC to aMCI and AD patients. (**a**,**b**) Comparison of fecal SCFAs and fecal secondary bile acids among the HC, aMCI, and AD groups. Note: Data are given as median with range and *P* values were calculated using one-way ANOVA or Kruskal–Wallis test. * *p* < 0.05; ** *p* < 0.01; *** *p* < 0.001. Abbreviations: SCFAs, short-chain fatty acids; DCA, deoxycholic acid; LCA, lithocholic acid; UDCA, ursodeoxycholic acid; nutriCA, nutriacholic acid; 12-ketoDCA, 12-ketodeoxycholic acid; iso-LCA, isolithocholic acid; ACA, allocholic acid; TDCA, taurodeoxycholic acid; TUDCA, tauroursodeoxycholic acid; GUDCA, glycoursodeoxycholic acid; GDCA, glycodeoxycholic acid; GLCA, glycolithocholic acid; BAs, bile acids; ANOVA, analysis of variance.

The targeted metabolomics profiling of SCFAs based on GC-MS was performed, which were the key gut-derived metabolites. In total, eight SCFAs were detected in the feces of all study subjects (Figure S2). Compared with HC, seven SCFAs showed a progressively decreased trend from aMCI to AD, six of the seven SCFAs were significantly lower in both aMCI and AD (*p* < 0.05), and the valeric acid was markedly decreased in AD (*p* < 0.05) (Figure 3a). Interestingly, when adjusting the observed significance level for multiple comparisons, five SCFAs including formic acid, acetic acid, propanoic acid, 2-methylbutyric acid, and isovaleric acid showed a significant difference between aMCI and AD patients (*p* < 0.05) (Table S7). Together, these results show that fecal SCFAs were changed associated with the disease progression of AD.

### 3.4. Perturbation of Fecal Bile Acids in AD Patients

Due to the low relative abundance of fecal BAs, the targeted metabolomics were conducted to examine the BAs metabolism. A total of thirty fecal BAs were profiled and quantitatively examined (Figure S3). Here, a total of eighteen primary (Figure S4a) and secondary BAs (Figure 3b) were detected among all subjects of the cohort. Of note, only the cytotoxic lithocholic acid (LCA) [33,34,35] showed a gradually decreased trend from HC to aMCI and AD, and a significant reduction in AD when compared with HC subjects (*p* = 0.039) (Figure 3b), while the multiple comparison correction results showed no significant difference in LCA between AD and HC (*p* = 0.126). There was no difference in the ratio of lithocholicacid (LCA) to chenodeoxycholic acid (CDCA), total LCA to total CDCA, total primary BAs, total secondary BAs and the ratio of total secondary to primary BAs in AD (Figure S4b–e). 

### 3.5. Association between the Disturbed Microbes of AD and Fecal Metabolites

**Figure 4:**
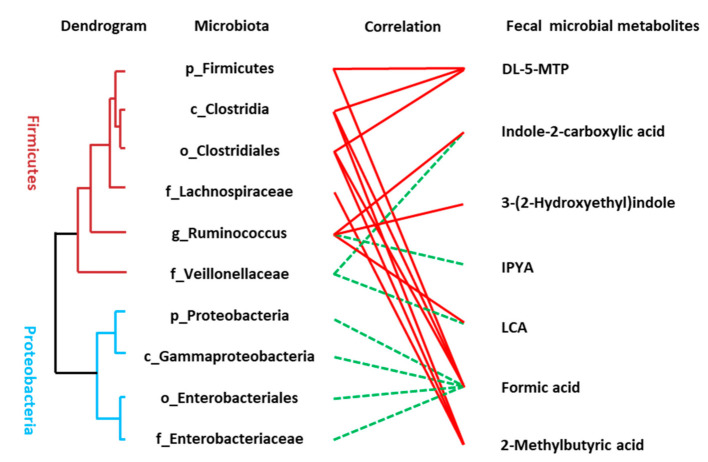
Linkage between altered microbes and differential microbial metabolites of AD patients. Correlation analysis between the altered microbiota and fecal metabolites were conducted using Spearman rank correlation or Pearson correlation analysis (red line means significantly positive correlation and green line means significantly negative correlation). The hierarchical cluster of gut microbiota was achieved based on the correlation coefficient of the relative abundance.

The Spearman correlation analysis was used to analyze the association between disordered microbes (Table S8) and the perturbed microbial signatures of AD including metabolites of the tryptophan pathway, SCFAs, and LCA. A significant linkage between the altered microbiota and metabolic perturbation of AD was presented (Figure 4). Many of the identified compounds are well known gut microbial metabolites such as DL-5-MTP, IPYA, LCA, and SCFAs. Some microbial members only revealed a single metabolite association, for example, the connection between *Lachnospiraceae* and 2-methylbutyric acid and some members had multiple connectivities, for example, *Clostridia* was statistically linked with the presence of three fecal metabolites including DL-5-MTP, formic acid, and 2-methylbutyric acid. 

Due to the remarkably altered concentration of SCFAs in feces of AD patients, we compared the reported 15 SCFA-producing bacteria [36,37,38] between HC, aMCI, and AD patients. As shown in Figure S5, *Clostridia*, with the capability of producing SCFAs like acetate, proprionate, butyratem and its phylum Firmicutes as well as the *Clostridiales*, *Ruminococcaceae*, *Ruminococcus* at order, family and genus levels, were significantly decreased in AD patients compared with aMCI or HC, while the propionate-producing *Bacteroides* was increased in aMCI patients compared with AD and HC (Figure S5). The consistent results of SCFA-producing bacteria and fecal SCFA concentration suggested that the decreased clusters of bacteria in Firmicutes, *Clostridia*, and *Clostridiales* of AD patients might contribute to the reduction of SCFAs. 

### 3.6. Serum Levels of Endotoxin Prone to Increase in AD

Given the role of pro-inflammatory lipopolysaccharide (LPS) in cognitive impairment [39], the circulating LPS level was measured. There was an increasing trend in the serum LPS of pre-onset aMCI (aMCI vs. HC, *p* = 0.210) and AD (AD vs. HC, *p* = 0.557) patients compared with HC individuals (Figure S6). These results exhibited a prone “leaky gut” in patients with both aMCI and AD. 

### 3.7. Association between the Altered Metabolites of AD and Cognitive Impairment

**Figure 5:**
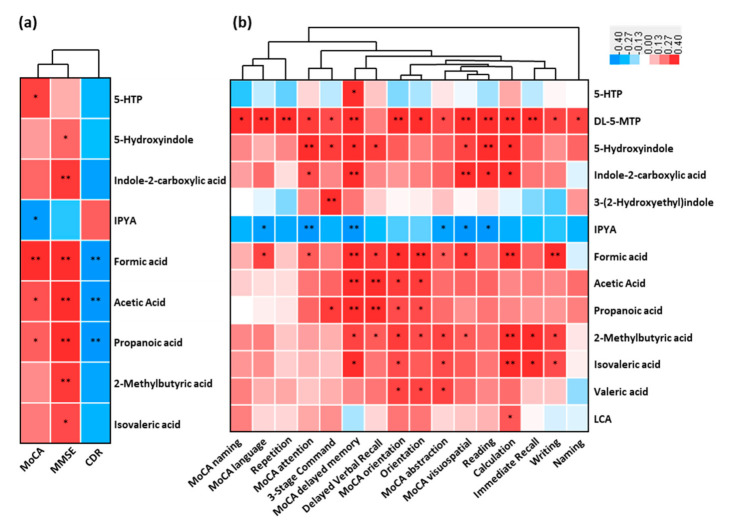
Linkage between altered metabolites of AD and cognitive impairment. Heat maps of Spearman rank correlation between the altered fecal metabolites including tryptophan metabolites, SCFAs, and bile acids, and (**a**) clinical parameters reflecting cognition function and disease severity, and (**b**) sub items in MoCA (naming, language, attention, delayed memory, orientation, abstraction and visuospatial) and MMSE sub domains (repetition, 3-stage command, delayed verbal recall, orientation, reading, calculation, immediate recall, writing and naming). Note: * *p* < 0.05; ** *p* < 0.01. Red means positive correlation and blue means negative. Abbreviations: MoCA, Montreal Cognitive Assessment; MMSE, Mini-Mental State Examination; CDR, Clinical Dementia Rating.

The association between the fecal altered microbial signatures and parameters reflecting disease severity (CDR), cognitive function (MMSE and MoCA) and almost all the subdomains were shown (Figure 5a,b). For example, the increased cognitive impairment (MoCA) positively correlated with the reduced serotonin (5-HTP) and SCFAs, while was negatively correlated to the increased indole-3-pyruvic acid of AD (*p* < 0.05). These decreased SCFAs were negatively associated with increased CDR (*p* < 0.05). Indole derivatives (5-hydroxyindole and indole-2-carboxylic acid) and SCFAs (including 2-methylbutyric acid and isovaleric acid) were positively associated with cognitive impairment (MMSE) (*p* < 0.05). Furthermore, a significant correlation existed between these fecal microbial signatures of AD and the cognitive impairment (almost all the subdomains of MoCA and MMSE) (Figure 5b). These results showed that the perturbed microbial signatures of AD correlated with MoCA and MMSE items of cognitive impairment. 

### 3.8. Classification of AD from aMCI and HC by Fecal Microbial Signatures

**Figure 6:**
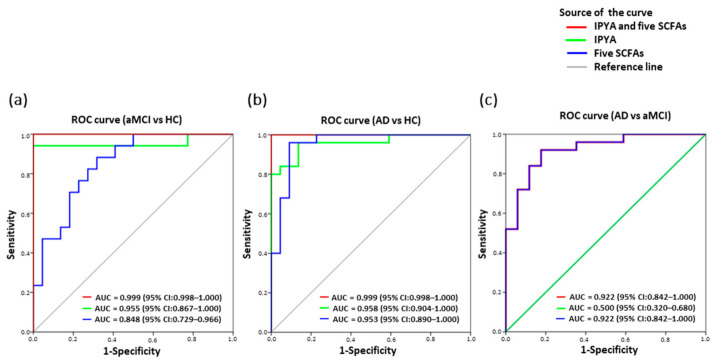
Classification and prediction of AD patients using the panel of six fecal microbial metabolites. Predictive models based on IPYA of tryptophan metabolites and five SCFAs (formic acid, acetic acid, propanoic acid, 2-methylbutyric acid and isovaleric acid) were built by performing binary multivariable logistic regression analysis. ROC curves were calculated for discriminating (**a**) aMCI from HC, (**b**) AD from HC, and (**c**) AD from aMCI. Abbreviations: ROC, receiver operating characteristic; AUC, area under the ROC curve; CI, confidence interval.

Among the fourteen fecal differentiating microbial metabolites of AD, a panel of six biomarkers including IPYA and five SCFAs effectively discriminated aMCI and AD from HC based on ROC curve analysis (aMCI vs. HC, AUC = 0.999, *p* = 0.000; AD vs. HC, AUC = 0.999, *p* = 0.000) (Figure 6a,b). Notably, IPYA accurately classified aMCI (AUC = 0.955, *p* = 0.000) and AD (AUC = 0.958, *p* = 0.000) from HC (Figure 6a,b; Table S9). Additionally, five SCFAs discriminated aMCI from AD (AUC = 0.922, *p* = 0.000) and HC (AUC = 0.848, *p* = 0.000) as well as AD from HC (AUC = 0.953, *p* = 0.000) (Figure 6a–c).

## 4. Discussion

Previous studies were focused on serum, CSF, and tissue metabolomics profiles of AD progression. However, our previous study found a great contribution of IM in AD progression and no fecal metabolomics profiles had been previously investigated. Thus, studies to explore whether fecal microbiota-metabolomics signatures could serve as new noninvasive biomarkers to differentiate AD patients in crucial stages including pre-onset aMCI and early AD dementia can greatly benefit early AD detection and intervention. By integrating high-throughput UPLC/MS and GC/MS-based metabolomics studies of fecal metabolites, the fecal metabolic dysregulation of AD pointed to tryptophan, SCFAs, and BAs pathways, which linked with gut dysbiosis and cognitive impairment. Moreover, the study showed that six microbial signatures, especially IPYA, could accurately classify both aMCI and AD from HC. Our study presented the fecal microbial signatures of AD, providing novel biomarkers and a therapeutic strategy for AD progression. 

The IM is a crucial player in human physiology. The dialogue between the microbiota and host are mediated by metabolites that are either produced by the microbiota or derived from the transformation of environmental or host molecules [7,8]. Here, we found that the distinct fecal metabolomics of AD including pre-onset stage aMCI and AD dementia compared with the cognitively healthy status. The perturbation in three categories of metabolites including tryptophan, SCFAs, and bile acids were found in AD patients. These metabolites have been recognized as the essential array at the interface between microorganisms and the host [7]. It is widely accepted that tryptophan can serve as a key prerequisite that coordinates the gastrointestinal physiology and CNS function [7], which was achieved through regulation of indole derivatives, the kynurenine pathway, and serotonin synthesis [7]. Here, we found that the fecal metabolomics of AD were characterized with the significant perturbation of tryptophan metabolism, especially the indole derivatives and serotonin synthesis, and were significantly linked with intestinal dysbiosis and AD development. The serotonin in both enteric and CNS is a key element in the gut–brain axis, acting as a neurotransmitter [40]. The abnormal 5-HT synthesis caused by intestinal dysbiosis could affect the pathological process of AD [41]. In addition, the well-known AhR signaling of tryptophan metabolites constituting the interface of microbiota–gut–brain axes [42], could suppress pro-inflammatory cytokines in astrocytes [43] and microglia [42], and has the potential to participate in several brain diseases including AD [42]. Consistent with the reported tryptophan metabolites in human CSF [16], our findings added evidence that fecal tryptophan metabolism might be under the control of microbiota and the crosstalk of microbiota–host might play a role in the pathogenesis of AD. 

Interestingly, we first revealed that fecal IPYA was enriched in pre-onset aMCI and AD, and significantly linked with intestinal dysbiosis and AD development. Notably, fecal IPYA could classify AD and aMCI from normal cognitive HC. IPYA is one of the major precursor metabolites that can be converted into numerous AhR ligands [42]. However, we did not find coordinately enriched AhR ligands in the intestine of AD patients, suggesting that the metabolic conversion of IPYA to AhR ligands was suppressed in aMCI and AD. Although AhR signaling gained has considerable attention in the field of AD, there have been no reports of dysregulation of intestinal IPYA in AD. Taken together, our results demonstrate that fecal tryptophan perturbation in AD, especially IPYA, which might be the mediator of intestinal dysbiosis and participate in AD development, is therefore a potential target for preventing or treating AD from a therapeutic perspective.

Notably, we found that SCFAs had strong predictive ability for the conversion from aMCI to AD. SCFAs are known to beneficially maintain both intestinal barrier function [8,44], and protect the integrity of the blood–brain barrier (BBB) [45,46]. Importantly, SCFAs directly influence the immune response [47], cross the BBB, and drive the maturation and function of microglia cells [48,49,50], which are the main macrophages in brain parenchyma. Moreover, SCFAs exert their effects by regulating the secretion of gut hormones like glucagon­like peptide-1, and these hormones could improve neuroplasticity in the hippocampus [8]. Interestingly, the restoration of the intestinal SCFAs help prevent or ameliorate AD pathology [51]. More recently, a study reported that the modified Mediterranean-ketogenic diet modulated the IM and improved SCFAs in MCI patients associated with the improved AD biomarkers in CSF [48]. Thus, the decreased SCFAs in intestine might be crucial mediators between IM and AD.

In addition, we first found that the fecal LCA decreased in AD and pre-onset aMCI were correlated with cognitive impairment. As the most hydrophobic BAs, LCA was the secondary BA and could cross the BBB to exert a cytotoxic effect for the brain [13,33]. Since LCA is converted from CDCA via 7α-dehydroxylation by IM, the unchanged CDCA and ratio of LCA: CDCA in AD indicated the enhanced re-absorption of LCA into the circulating system and potentially impaired the CNS. In line with this finding, the increased LCA in AD was reported in both the serum of patients [52,53] and the brain tissue of AD transgenic mice [54], but was not found in other reported studies [13]. These inconsistent findings might be due to the different sample types and the uncontrolled effects of factors among cohorts from different geographical origins of western and eastern countries. Taken together, our findings add new clues for the role of gut-derived bile acid LCA in the pathogenesis of AD. This deserved further study in a larger cohort. 

We hypothesized that the gut dysbiosis associated with AD patients resulted in the dysregulation metabolism of tryptophan, SCFAs, and BAs. Furthermore, the perturbation of intestinal microbiota-metabolites could increase intestinal permeability, permit LPS translocation, or directly incur or aggravate neuroinflammation and neurotransmission disorders, and finally promote AD progression. 

Despite these exciting findings of AD, there were some limitations in this study. For instance, the diagnosis of AD was made according to the criteria of DSM-IV and guidelines of NINCDS-ADRDA [10]. Nevertheless, these criteria have been widely used for population-based studies and reported with quite high accuracy by the postmortem analysis [19]. Here, the recruited volunteers refused to perform the lumbar puncture for the CSF assay and positron emission tomography imaging, which have a low degree of acceptance in the elderly [55]. Second, this observational study failed to demonstrate the causality role of microbial metabolites in AD development. However, most of these metabolites were progressively altered with disease progression and significantly correlated with cognitive impairment, strongly indicating their critical role in AD. Further longitudinal studies in large cohorts are required to focus on more detailed periods and follow-up investigation to define their cause–effect relationship.

## 5. Conclusions

In the present study, the highly reproducible and high-throughput targeted and untargeted UPLC/GC-MS were used to profile the fecal metabolomics of pre-onset aMCI and AD, and revealed the altered fecal microbial signatures for AD. The perturbation of fecal microbiota-metabolomics pointed to the dysregulation pathways of tryptophan, SCFAs and bile acids. Intriguingly, fecal IPYA was first reported to discriminate both pre-onset aMCI and AD from HC, and five SCFAs discriminated AD from aMCI. Importantly, the best results for detecting AD and aMCI were achieved when IPYA was combined with five SCFA measurements. Thus, these findings provide the evidence that fecal microbial signatures could be noninvasive biomarkers for AD screening and management, and targeting IM or microbial metabolism might be a promising method for preventing and treating AD.

## Figures and Tables

**Table 1:** Demographics and clinical parameters of subjects in the Alzheimer’s disease (AD), amnestic mild cognitive impairment (aMCI), and normal cognition healthy control (HC) groups.

Characteristics	HC	aMCI	AD
	(n = 28)	(n = 22)	(n = 27)
Age (years)	74.25 ± 9.03	70.00 ± 11.33	74.15 ± 11.16
Sex (male/female)	14/14	9/13	15/12
Education (years)	9 (6–12)	9 (9–12)	9 (6–9)
BMI (kg/m^2^)	22.29 ± 2.18	22.68 ± 2.17	21.87 ± 1.15
Diabetes (%)	0 (0%)	2 (9.1%)	5 (18.7%)
Hypertension (%)	9 (32.1%)	9 (40.9%)	11 (40.7%)
Fasting glucose (mmol/L)	5.23 ± 0.69	6.37 ± 1.93	5.02 ± 0.54
Hemoglobin (g/L)	138.42 ± 17.85	142.19 ± 11.23	136.79 ± 11.38
Folic acid (ng/mL)	11.08 ± 5.16	8.97 ± 2.90	8.75 ± 3.66
Vitamin B12 (pg/mL)	484.00 (435.50–716.00)	510.00 (379.00–685.00)	383.50 (341.00–506.25)
TT4 (nmol/L)	107.05 ± 22.31	108.44 ± 19.74	103.50 ± 35.06
TT3 (nmol/L)	1.53 (1.43–1.92)	1.72 (1.44–1.89)	1.55 (1.38–1.75)
MMSE	29.00 (26.00–29.50)	27.00 (26.00–28.00)	18.00 (13.50–23.00) ***^###^
MoCA	26.00 (24.50–27.00)	22.00 (18.00–24.00) *	17.00 (14.50–19.00) ***^#^

Note: Data are given as the mean ± SD or median (IQR). * *p* < 0.05; *** *p* < 0.001 compared with HC and ^#^ *p* < 0.05; ^###^ *p* < 0.001 compared with aMCI group. Abbreviations: HC, normal cognition healthy control; aMCI, amnestic mild cognitive impairment; AD, Alzheimer’s disease; BMI, body mass index; TT3, total triiodothyronine; TT4, total thyroxine; MMSE, Mini-Mental State Examination; MoCA, Montreal Cognitive Assessment; SD, standard deviations; IQR, interquartile range.

**Table 2:** Sub item Montreal Cognitive Assessment (MoCA) scores of AD, aMCI, and HC groups.

MoCA Sub Item	HC	aMCI	AD
	(n = 28)	(n = 22)	(n = 27)
Visuospatial	5 (4.75–5)	4 (3–5)	3 (1.25–3.75) ***^#^
Naming	3 (3–3)	3 (2–3)	2 (2–3) **
Attention	3 (3–3)	3 (2–3)	1.5 (1–2.75) **^#^
Language	2.5 (2–3)	2 (1–2)	1 (0–1) ***^#^
Abstraction	1.5 (1–2)	0 (0–1)	0 (0–0) ***
Delayed recall	3.5 (3–5)	1 (0–3) **	0.5 (0–1) ***
Orientation	6 (5.75–6)	6 (5–6)	3 (3–5.75) **^##^

Note: Data are given as the median (IQR). ** *p* < 0.01; *** *p* < 0.001 compared with HC and ^#^ *p* < 0.05; ^##^ *p* < 0.01 compared with aMCI group. Abbreviations: HC, normal cognition healthy control; aMCI, amnestic mild cognitive impairment; AD, Alzheimer’s disease; MoCA, Montreal Cognitive Assessment; IQR, interquartile range.

## Data Availability

The sequence dataset of the fecal microbiota can be downloaded from the National Center for Biotechnology Information (NCBI) Bioproject database (PRJNA496408).

## Supplementary Materials

The following are available online at https://www.mdpi.com/2072-6643/13/1/228/s1 including Supplementary Materials and Methods, Supplementary Tables S1–S9, Supplementary Figures S1–S6.
