# Supplementary material for: Altered Gut Microbial Metabolites in Amnestic Mild Cognitive Impairment and Alzheimer’s Disease: Signals in Host–Microbe Interplay
Source: Nutrients. 2021 Jan 14;13(1):228. doi: 10.3390/nu13010228 (PMC7829997; doi:10.3390/nu13010228)
Supplement: Supplementary file 1 [file nutrients-13-00228-s001.pdf]

## 1. Supplementary Materials and Methods

*Study subjects*

The enrolled Alzheimer's disease (AD) patients in this study were diagnosed based on criteria of Diagnostic and Statistical Manual (DSM)-IV [1] and guidelines of the National Institute of Neurological and Communicative Disorders and the Stroke and Alzheimer Disease and Related Disorders Association (NINCDS-ADRDA) [2], including memory impairment more than 6 months reported by patient or informant, and medial temporal lobe atrophy. All enrolled AD patients with Clinical Dementia Rating (CDR) score at least 1 [3], and Mini-Mental State Examination (MMSE) scores $\leq$ 24 for patients at education level of junior school or above, $\leq$ 20 for education level of primary school, and $\leq$ 17 for illiteracy [4]. The enrolled amnestic mild cognitive impairment (aMCI) were diagnosed in a fashion similar to DSM-IV or the NINCDS-ADRDA criteria, including memory complaint usually evidenced by an informant and normal activities of daily living [5]. The aMCI patients enrolled with MMSE scores between 24 to 30 and a CDR score of 0.5. The normal cognition healthy controls (HC) were age- and sex- matched individuals with normal cognitive function, and most of them were patient's spouses with MMSE scores between 24 and 30 and CDR scores of 0. These criteria were amended for Chinese individuals [6].

Demographic information including age, years of education, and history of diabetes and hypertension were obtained during the enrollment visit. Each participant underwent a complete physical examination in our Memory Clinic of our Neurology Department. The body weight and height measurement were collected to calculate body mass index. For the neuropsychological assessments, the Montreal Cognitive Assessment (MoCA) test was applied, which is the most sensitive cognitive screening tool to differentiate mild cognitive impairment (MCI) from normal aging and composed of naming,

language, attention, delayed memory, orientation, abstraction and visuospatial. And MMSE was used to quantify cognitive function, including repetition, 3-stage command, delayed verbal recall, orientation, reading, calculation, immediate recall, writing and naming. The severity of dementia was evaluated by CDR scale. The neuroimaging examinations were conducted using magnetic resonance imaging (MRI). Additionally, the fasting serum samples were collected and biochemical parameters including hemoglobin, folic acid, vitamin B12, total triiodothyronine and total thyroxine were examined to exclude other causes of cognitive impairment.

The exclusion criteria were as follows: 1) other causes of dementia and neuropsychiatric disorders like schizophrenia, schizoaffective disorder or primary affective disorder, etc.; 2) auditory, visual or motor deficits might interfere the cognitive assessment results; 3) irritable bowel syndrome and inflammatory bowel disease in the last year; 4) serious primary disease in heart, brain, liver and other important systems; 5) antibiotics, probiotics, prebiotics or symbiotic treatment within two months [4].

*The bacterial 16S rRNA gene Miseq sequencing*

Fresh fecal samples were collected from 93 subjects (28 HC, 32 aMCI and 33 AD) using sterile collection container. The fecal samples were aliquoted to 200 mg in 2 ml Eppendorf tube on ice immediately, and stored at -80 ℃ until analysis. The composition of intestinal microbiota was determined by 16S rRNA gene Miseq sequencing as previously described [4]. Briefly, the microbial genomic DNA was extracted from fecal samples using DNA extraction kit (QIAGEN, Hilden, Germany) [7, 8]. The concentration of the extracted DNA was determined by Nanodrop 1000 spectrophotometer (Thermo Fisher Scientific, USA) and the DNA quality was evaluated using 1.0% agarose gel electrophoresis. Then the bacterial genomic DNA was used as template for PCR amplification of 16S rRNA V3-V4 region with primer pair

(F: 5’-CAAGCAGAAGACGGCATACGAGATGTGACTGGAGTTCAGACGTGTGCTCTTCCGATCT-3’; R: 5’-AATGATACGGCGACCACCGAGATCTACACTCTTTCCCTACACGACGCTCTTCCGATCT-3’). Next, equimolar concentrations of PCR products were pooled, and sequenced in Illumina® MiSeq platform. Clean reads with 97% similarity were clustered into operational taxonomic units (OTU) and the OTU tables with associated Greengenes identifiers were produced from QIIME software [9, 10].

*High-throughput fecal untargeted metabolomics profiling*

*Chemical Reagents.* HPLC grade methanol and ultrahigh quality water were purchased from Thermo Fisher Scientific (USA).

*Sample Preparation.* The fresh fecal samples were collected and stored at -80 ℃ until analysis. The samples were extracted by methanol at a ratio of 3 mL/g [11], and ceramic beads (1 mm, OMNI, USA) were added for homogenizing (Omni International, USA). Then, the mixtures were centrifuged twice (12,000 rpm, 10 min, Eppendorf, Germany) and the supernatant was collected and filtered using 0.22 μm syringe filters (Millipore, USA). An equal volumes of all extracted samples were pooled as the QC sample.

*Metabolomics profiling analysis.* In this study, the Dionex UltiMate 3000 RS system coupled with Q Exactive HF-X mass spectrometry (MS) (Thermo Fisher Scientific, USA) was applied for metabolite separation and MS detection [12]. The liquid chromatographic separation was performed by a Hypersil Gold C-18 column (2.1×100mm, 1.9 μm, Thermo Fisher Scientific, USA). And the mobile phase consisted of A (water with 0.1% formic acid, v/v) and B (methanol) under ionization-positive (ESI +) mode, and A (water) and B (methanol) were used under electrospray ionization-negative (ESI -) mode. The elution gradients were set as follows: 2% of B during 0- 0.5

min, 2%- 40% B during 0.5- 8 min, 40%- 98% B during 8- 12 min, 98% B during 12-14 min, and 2% of B at last 2.5 min. MS detection was conducted by Q Exactive HF-X MS with heated-ESI-II (HESI-II) ion source (Thermo Fisher Scientific, USA) as previously described [12]. The acquisition mode was a full MS with a m/z range 70-1050 followed by data-dependent $MS^2$ (dd-$MS^2$). The resolution was set at 60,000 and 15,000 for full MS and dd-$MS^2$, respectively. The $MS^2$ spectrometry data were acquired with the collision energy of 20, 40 and 60 eV.

In order to delete the confounding factors during the experiment, the blank sample (100% HPLC-grade water) was run with the sample simultaneously. Ten QC samples were run before sample analyzing to equilibrate the detection system, and one QC sample was run every ten samples during sample processing to monitor the stability of the acquisition system [13].

*Data processing.* The data processing was performed in Compound Discoverer 3.1 software (Thermo Fisher Scientific, USA) according to the manufacturer's user guideline. The multivariate statistical analysis including principle component analysis (PCA) and partial least-squares-latent structure discriminate analysis (PLS-DA) were performed using SIMCA-P 13.0 (Umetrics AB, Sweden). And the univariate analysis was conducted by one-way ANOVA or Kruskal-Wallis test in SPSS software (version 16.0, SPSS Inc., USA) and GraphPad Prism 6 (GraphPad Inc., USA). To identify potential biomarkers for AD, the relative abundance of metabolites were calculated by assigning the total peak area of the metabolic profiles from one sample to $10^7$, and receiver operating characteristic (ROC) analysis based on the relative abundance of differential metabolites were performed.

*Targeted profiles of fecal short-chain fatty acids*

*Standard chemicals and reagents in SCFAs detection.* Totally fifteen short-chain fatty

acids (SCFAs) standards were quantitative examined. The standard compounds of formic acid and acetic acid were purchased from Thermo Fisher Scientific (USA), 3-methylvaleric acid was purchased from Tokyo Chemical Industry Co., Ltd (Japan), and the other twelve SCFAs standards including propanoic acid, isobutyric acid, butyric acid, 2-methylbutyric acid, isovaleric acid, valeric acid, 4-methylvaleric acid, hexanoic acid, 2-methylhexanoic acid, 4-methylhexanoic acid, heptanoic acid and octanoic acid, were purchased from Sigma-Aldrich Inc. (USA). The HPLC grade chloroform and NaOH were purchased from Sinopharm Chemical Reagent Co., Ltd. (China), and isobutanol and isobutyl chloroformate were provided by Alfa Aesar (USA) and Amethyst Chemicals (China), respectively.

*Sample Preparation.* The fecal sample from each subject were collected and derivatized as previously described [14, 15]. Briefly, samples were prepared by mixing with 10% isobutanol, then ceramic beads were added for homogenizing (50 Hz, 30 s). After centrifugation (12000rpm, 5min), the supernatant was obtained and chloroform were added to remove lipophilic compounds. For chloroformate derivatization, the esterification was performed using isobutanol and isobutyl chloroformate, and NaOH and pyridine were added as base and catalyst, respectively. One boiling stone (Acros Organics, USA) was added to avoid bumping. Finally, hexane was added and centrifuged, and the upper hexane-isobutanol phase was transferred into a gas chromatography (GC) vial for detection.

*GC-MS Analysis.* SCFAs quantitative profiling were performed on Agilent 7890B GC equipped with MS (5977, Agilent Technologies, USA). As previously reported by Takeshi Furuhashi et al. with mild modification [14], HP-5MS 30 m, 0.25 mm, 0.25 μm (Agilent Technologies, USA) was equipped as a GC column. The oven temperature was set as follows: The initial oven temperature was kept at 50 ℃ for 5 min, then the

temperature was ramped to 150 ℃ at a rate of 5 ℃/min, next rise to 325 ℃ at a rate of 40 ℃/min, finally kept at 325 ℃ for 1 min. The acquisition mode of MS was single ion monitoring (SIM). The chosen fragments for quantification of SCFAs were listed in **Table S2**. To quantify SCFAs, the peak areas were extracted using Mass Hunter software (Agilent Technologies, USA).

*Targeted profiles of fecal bile acids*

*Standard chemicals and reagents in bile acids detection.* HPLC grade methanol, acetonitrile and formic acid were purchased from Sigma-Aldrich Inc. (USA). Seven deuterated BAs were used as internal standards (**Table S3**). And thirty bile acids (BAs) standard compounds were purchased from Steraloids Inc (USA) or Toronto Research Chemicals Inc. (Canada) (**Table S4**).

*Sample Preparation.* Fecal samples were prepared as previously described [16]. Briefly, fecal samples were mixed with extraction solvent (methanol: $H_2O$ = 2: l, 0.005% formic acid, v/v/v) containing internal standards. After homogenization, the mixture experienced rapid freeze- thaw cycles three times in liquid nitrogen, followed by homogenization (50Hz, 30s) and centrifugation (12000 rpm, 10 min). The supernatant was filtered through a 0.22 μm filter (Nylon-66 syringe filters, Tianjin, China).

*UPLC-MS Analysis.* The BAs were determined by an Agilent 1290 ultraperformance liquid chromatography (UPLC) coupled with an Agilent 6470 triple quadrupole mass spectrometer (MS) (Agilent Technologies, USA). Sample separation was achieved by Kinetex® Core-Shell 2.6 μm C18 column (100 × 2.1mm, 2.6 μm, Phenomenex, USA). The BAs profiles were analyzed as previously described with minor modifications [17]. The mobile phase consisted of A (water with 0.005% formic acid, v/v) and B (acetonitrile with 0.005% formic acid, v/v). The eluent gradients were set as follows:

23%- 33% of B during 0- 2 min, 33%- 34% B during 2- 6 min, 34%- 70% B during 11- 11.01 min, 70%- 95% B during 11.01-15 min, and 95% of B at last 5 min. And the acquisition mode of MS detection was multiple reactions monitoring (MRM). Data were collected using Mass Hunter software (Agilent Technologies, USA). The BAs were identified by referring to the retention time and ion pairs of standard chemicals (**Table S5**) and quantified by the internal standard calibration curves.

*Measurement of circulating lipopolysaccharide level*

The serum lipopolysaccharide (LPS) was measured using limulus amoebocyte lysate (LAL) chromogenic endpoint assay (Hycult Biotech, Uden, Netherlands) in 48 subjects (AD, n = 12; aMCI, n = 27; HC, n = 9) [18]. The serum samples were diluted 1:3 with endotoxin-free water and heated at 75 ℃ for 5 minutes in a water bath to neutralize the endotoxin inhibiting compounds. Then, the LAL reagent was added and incubated with sample for 20 min at 25 ℃. Finally, the reaction was terminated by adding the stop solution and measured by spectrophotometer (Biotek, Vermont, USA).

## 2. Supplementary Tables

**Supplementary Table S1. Mass list library of twenty tryptophan metabolites.**

| **Name** | **Formula** |
|---|---|
| Indole-3-Pyruvic acid | C11H9NO3 |
| Indole-3-Lactic Acid | C11H11NO3 |
| Indole Acrylic Acid | C11H9NO2 |
| Indole-3-Propionic Acid | C11H11NO2 |
| Indole-3-Acetamide | C10H10N2O |
| Indole-3-Acetic acid | C10H9NO2 |
| Indole-3-Aldehyde | C9H7NO |
| Indole | C8H7N |
| Kynurenine | C10H12N2O3 |
| Kynurenic acid | C10H7NO3 |
| 3-Hydroxykynurenine | C10H12N2O4 |
| 3-Hydroxyanthranilic Acid | C7H7NO3 |
| Quinolinic acid | C7H5NO4 |
| Xanthurenic acid | C10H7NO4 |
| Picolinic acid | C6H5NO2 |
| 5-Hydroxytryptophan | C11H12N2O3 |
| Serotonin | C10H12N2O |
| N-Acetylserotonin | C12H14N2O2 |
| Melatonin | C13H16N2O2 |
| 5-Hydroxyindole acetic acid | C10H9NO3 |

**Supplementary Table S2. The retention time and fragments of fifteen short-chain fatty acids standard compounds.**

| SCFAs | RT (min) | Fragments |
|---|---|---|
| Formic acid | 2.395 | 56, 43, 41 |
| Acetic acid | 3.554 | 56, 43, 73 |
| Propanoic acid | 6.162 | 57, 29, 87 |
| Isobutyric acid | 7.813 | 71, 43, 56 |
| Butyric acid | 9.301 | 71, 56, 43 |
| 2-Methylbutyric acid | 10.964 | 85, 57, 103 |
| Isovaleric acid | 11.090 | 85, 57, 29 |
| Valeric acid | 12.716 | 85, 57,103 |
| 3-Methylvaleric acid | 14.583 | 99, 29, 56 |
| 4-Methylvaleric acid | 14.758 | 99, 56, 81 |
| Hexanoic acid | 15.854 | 99, 56, 29 |
| 2-Methylhexanoic acid | 16.788 | 113, 131, 85 |
| 4-Methylhexanoic acid | 17.940 | 113, 131, 56 |
| Heptanoic acid | 18.778 | 113, 56, 131 |
| Octanoic acid | 21.488 | 127, 57, 145 |

**Supplementary Table S3. The seven internal standards of bile acids.**

| Internal Standards | Ion pairs (m/z) | Polarity |
|---|---|---|
| TCA-d | 518.4/80 | negative |
| GCA-d | 468.3/74.1 | negative |
| CA-d | 411.3/347.3 | negative |
| GCDCA-d | 452.3/74.1 | negative |
| CDCA-d | 395.3/395.3 | negative |
| DCA-d | 395.4/394.3 | negative |
| LCA-d | 379.3/379.3 | negative |

Abbreviations: TCA, taurocholic acid; GCA, glycocholic acid; CA, cholic acid; GCDCA, glycochenodeoxycholic acid; CDCA, chenodeoxycholic acid; DCA, deoxycholic acid; LCA, lithocholic acid.

**Supplementary Table S4. List of thirty bile acid standard compounds.**

| Abbreviation | Full name | Company |
|---|---|---|
| T-α-MCA | Tauro-Alpha-Muricholic Acid | Steraloids |
| T-β-MCA | Tauro-Beta-Muricholic Acid | Steraloids |
| THCA | Taurohyocholic Acid | Steraloids |
| TUDCA | Tauroursodeoxycholic Acid | Steraloids |
| TCA | Taurocholic Acid | Steraloids |
| GHCA | Glycohyocholic Acid | Steraloids |
| GCA | Glycocholic Acid | Steraloids |
| ω-MCA | Omega-Muricholic Acid | Steraloids |
| GUDCA | Glycoursodeoxycholic Acid | Steraloids |
| GHDCA | Glycohyodeoxycholic Acid | Steraloids |
| α-MCA | Alpha-Muricholic Acid | Steraloids |
| β-MCA | Beta-Muricholic Acid | Steraloids |
| TCDCA | Taurochenodeoxycholic Acid | Steraloids |
| HCA | Hyocholic Acid | Steraloids |
| TDCA | Taurodeoxycholic Acid | Steraloids |
| CA | Cholic Acid | Steraloids |
| GCDCA | GlycochenodeoxycholicAcid | Steraloids |
| UDCA | Ursodeoxycholic Acid | Steraloids |
| HDCA | Hyodeoxycholic Acid | Steraloids |
| GDCA | Glycodeoxycholic Acid | Steraloids |
| nutriCA | Nutriacholic Acid | Steraloids |
| 12-ketoDCA | 12-Ketodeoxycholic Acid | Steraloids |

| | | |
|---|---|---|
| TLCA | Taurolithocholic Acid | Steraloids |
| CDCA | Chenodeoxycholic Acid | Steraloids |
| DCA | Deoxycholic Acid | Steraloids |
| GLCA | Glycolithocholic Acid | Steraloids |
| iso-DCA | Isodeoxycholic Acid | Steraloids |
| iso-LCA | Isolithocholic Acid | Steraloids |
| LCA | Lithocholic Acid | Steraloids |
| ACA | Allocholic Acid | Toronto Research Chemicals |

**Supplementary Table S5. Ion pairs for bile acids qualification and quantification analysis.**

| Bile acids | Qualitative ion pair (m/z) | Quantitative ion pair (m/z) | Polarity |
|---|---|---|---|
| T-α-MCA | 514.3/124 | 514.3/107 | negative |
| T-β-MCA | 514.3/124 | 514.3/80 | negative |
| THCA | 514.3/124 | 514.3/80 | negative |
| TUDCA | 498.3/124 | 498.3/80 | negative |
| TCA | 514.3/80 | 514.3/124 | negative |
| GHCA | 464.6/354.3 | 464.6/74.1 | negative |
| GCA | 464.3/386.3 | 464.3/74.1 | negative |
| ω-MCA | 407.3/387.3 | 407.3/405.3 | negative |
| GUDCA | 448.4/386.3 | 448.4/74.1 | negative |
| GHDCA | 448.4/386.3 | 448.4/74.1 | negative |
| α-MCA | 407.3/387.3 | 407.3/405.3 | negative |
| β-MCA | 407.3/371.3 | 407.3/407.3 | negative |
| TCDCA | 498.3/124 | 498.3/80 | negative |
| HCA | 407.3/389.3 | 407.3/407.3 | negative |
| TDCA | 498.3/80 | 498.3/124 | negative |
| ACA | 407.3/363.3 | 407.3/361.3 | negative |
| CA | 407.3/289.2 | 407.3/343.3 | negative |
| GCDCA | 448.3/386.3 | 448.3/74.1 | negative |
| UDCA | 391.3/373.5 | 391.3/391.3 | negative |
| HDCA | 391.3/373.3 | 391.3/391.3 | negative |
| GDCA | 448.4/402.3 | 448.4/74.1 | negative |
| nutriCA | 389.3/343.4 | 389.3/389.5 | negative |
| 12-ketoDCA | 389.5/343.3 | 389.5/389.5 | negative |
| TLCA | 482.3/124 | 482.3/80 | negative |
| CDCA | 391.3/373.3 | 391.3/391.3 | negative |
| DCA | 391.3/343.3 | 391.3/345.3 | negative |
| GLCA | 432.3/386.3 | 432.3/74.1 | negative |
| iso-DCA | 391.3/327.3 | 391.3/345.3 | negative |
| iso-LCA | 359.3/81.1 | 375.3/375.3 | negative/positive |
| LCA | 359.3/81.1 | 375.3/375.3 | negative/positive |

Abbreviations: T-α-MCA, tauro-alpha-muricholic acid; T-β-MCA, tauro-beta-muricholic acid; THCA, taurohyocholic acid; TUDCA, tauroursodeoxycholic acid; TCA, taurocholic acid; GHCA, glycohyocholic acid; GCA, glycocholic acid; ω-MCA, omega-muricholic acid; GUDCA, glycoursodeoxycholic acid; GHDCA , glycohyodeoxycholic acid; α-MCA, alpha-muricholic acid; β-MCA, beta-muricholic acid; TCDCA, taurochenodeoxycholic acid; HCA, hyocholic acid; TDCA, taurodeoxycholic acid; ACA, allocholic acid; CA, cholic acid; GCDCA, glycochenodeoxycholic acid; UDCA, ursodeoxycholic acid; HDCA, hyodeoxycholic acid; GDCA, glycodeoxycholic acid; nutriCA, nutriacholic acid; 12-ketoDCA, 12-ketodeoxycholic acid; TLCA, taurolithocholic acid; CDCA, chenodeoxycholic acid; DCA, deoxycholic acid; GLCA, glycolithocholic acid; iso-DCA, isodeoxycholic acid; iso-LCA, isolithocholic acid; LCA, lithocholic acid.

**Supplementary Table S6. Differential tryptophan metabolites in fecal metabolites among AD, aMCI and HC groups.**

| RT_m/z | Adduct | Identified results | **fold change** | | | ***P* value** | | | **q value** | | |
|---|---|---|---|---|---|---|---|---|---|---|---|
| | | | AD vs HC | aMCI vs HC | AD vs aMCI | AD vs HC | aMCI vs HC | AD vs aMCI | AD vs HC | aMCI vs HC | AD vs aMCI |
| 8.062_233.09299 | M-H | DL-5-Methoxytryptophan | 0.005 | 0.006 | 0.745 | 0.000 | 0.000 | - | 0.000 | 0.000 | - |
| 6.741_132.04463 | M-H | 5-Hydroxyindole | 0.012 | 0.013 | 0.908 | 0.000 | 0.000 | - | 0.000 | 0.000 | - |
| 9.720_202.05029 | M-H | Indole-3-pyruvic acid | 21.021 | 17.986 | 1.169 | 0.000 | 0.017 | - | 0.000 | 0.000 | - |
| 10.828_162.02495 | M-H | Indole-2-carboxylic acid | 0.066 | 0.082 | 0.808 | 0.000 | 0.000 | - | 0.000 | 0.000 | - |
| 7.475_160.03954 | M-H | 3-(2-Hydroxyethyl)indole | 0.104 | 0.086 | 1.213 | 0.000 | 0.001 | - | 0.000 | 0.000 | - |

Note: Fold change was calculated according to ratio of group area. *P* values were determined by one-way ANOVA or Kruskal-Wallis test. And multiple comparison corrections were conducted using False Discovery Rate (FDR). Abbreviations: HC, normal cognition healthy control; aMCI, amnestic mild cognitive impairment; AD, Alzheimer's disease.

**Supplementary Table S7. Comparison of eight detected SCFAs in feces among AD, aMCI and HC groups.**

| Metabolites | *P* value | | | q value | | |
|---|---|---|---|---|---|---|
| | HC vs aMCI | HC vs AD | aMCI vs AD | HC vs aMCI | HC vs AD | aMCI vs AD |
| Formic acid | 0.003 | 0.000 | - | 0.015 | 0.000 | 0.019 |
| Acetic Acid | 0.020 | 0.000 | - | 0.044 | 0.000 | 0.005 |
| Propanoic acid | 0.012 | 0.001 | - | 0.043 | 0.000 | 0.043 |
| Butyric acid | 0.019 | 0.005 | - | - | 0.025 | - |
| 2-Methylbutyric acid | 0.012 | 0.000 | - | 0.035 | 0.000 | 0.035 |
| Isovaleric acid | 0.013 | 0.000 | - | 0.011 | 0.000 | 0.020 |
| Valeric acid | - | 0.016 | - | - | 0.024 | - |
| Hexanoic acid | - | - | - | - | - | - |

Note: *P* values were determined by one-way ANOVA or Kruskal-Wallis test. And multiple comparison corrections were conducted using False Discovery Rate (FDR). Abbreviations: SCFAs, short-chain fatty acids; HC, normal cognition healthy control; aMCI, amnestic mild cognitive impairment; AD, Alzheimer’s disease.

**Supplementary Table S8. The relative abundance of eighteen differential microbiota among AD, aMCI, and HC groups.**

| Microbes | HC<br>n= 28 | MCI<br>n= 32 | AD<br>n= 33 | aMCI vs HC<br>*P* value | AD vs HC<br>*P* value | AD vs aMCI<br>*P* value |
|---|---|---|---|---|---|---|
| p_Firmicutes | 0.737 (0.565-0.882) | 0.638 (0.417-0.756) | 0.601 (0.247-0.708) | - | 0.008 | - |
| p_Proteobacteria | 0.047 (0.013-0.332) | 0.059 (0.019-0.288) | 0.195 (0.062-0.710) | - | 0.024 | 0.029 |
| p_Bacteroidetes | 0.038 (0.014-0.112) | 0.132 (0.056-0.312) | 0.028 (0.011-0.093) | 0.018 | - | 0.001 |
| c_*Clostridia* | 0.658 (0.448-0.842) | 0.563 (0.396-0.700) | 0.418 (0.190-0.623) | - | 0.001 | 0.029 |
| c_*Gammaproteobacteria* | 0.038 (0.007-0.323) | 0.041 (0.008-0.278) | 0.182 (0.053-0.706) | - | 0.032 | 0.025 |
| c_*Bacteroidia* | 0.038 (0.014-0.112) | 0.132 (0.056-0.312) | 0.028 (0.011-0.093) | 0.018 | - | 0.001 |
| o_*Clostridiales* | 0.658 (0.448-0.842) | 0.563 (0.396-0.700) | 0.418 (0.190-0.623) | - | 0.001 | 0.029 |
| o_*Enterobacteriales* | 0.024 (0.006-0.205) | 0.034 (0.008-0.267) | 0.177 (0.036-0.703) | - | 0.015 | 0.038 |
| o_*Bacteroidales* | 0.038 (0.014-0.112) | 0.132 (0.056-0.312) | 0.028 (0.011-0.093) | 0.018 | - | 0.001 |
| f_*Clostridiaceae* | 0.039 (0.018-0.071) | 0.021 (0.009-0.040) | 0.015 (0.003-0.037) | 0.015 | 0.002 | - |

| | | | | | | |
|---|---|---|---|---|---|---|
| f_*Lachnospiraceae* | 0.193 (0.111-0.356) | 0.143 (0.083-0.231) | 0.143 (0.058-0.201) | 0.021 | 0.006 | - |
| f_*Ruminococcaceae* | 0.142 (0.083-0.225) | 0.161 (0.093-0.240) | 0.081 (0.040-0.138) | - | 0.019 | 0.001 |
| f_*Veillonellaceae* | 0.024 (0.010-0.059) | 0.076 (0.031-0.148) | 0.035 (0.009-0.105) | 0.018 | - | - |
| f_*Enterobacteriaceae* | 0.024 (0.006-0.205) | 0.034 (0.008-0.267) | 0.177 (0.036-0.703) | - | 0.015 | 0.038 |
| f_*Bacteroidaceae* | 0.021 (0.009-0.049) | 0.063 (0.017-0.130) | 0.015 (0.003-0.045) | 0.032 | - | 0.002 |
| g_*Blautia* | 0.080 (0.043-0.192) | 0.063 (0.024-0.091) | 0.053 (0.016-0.114) | 0.007 | 0.024 | - |
| g_*Ruminococcus* | 0.031(0.005-0.066) | 0.011(0.002-0.024) | 0.006(0.002-0.015) | - | 0.013 | - |
| g_*Bacteroides* | 0.021(0.009-0.049) | 0.063(0.017-0.130) | 0.015(0.003-0.045) | 0.032 | - | 0.002 |

Note: Data are given as median (IQR). *P* values were determined using a one-way ANOVA or Kruskal-Wallis test. Abbreviations: HC, normal cognition healthy control; aMCI, amnestic mild cognitive impairment; AD, Alzheimer’s disease; IQR, interquartile range; p, phylum; c, class; o, order; f, family; g, genus.

**Supplementary Table S9. Predictive models based on differential microbial metabolite for classification and prediction of AD.**

| Models | Metabolite | AUC | *P*-value | CI |
|---|---|---|---|---|
| aMCI vs. HC | Indole-3-pyruvic acid | 0.955 | 0.000 | 0.867-1.000 |
| | 5-Hydroxytryptophan | 0.294 | 0.029 | 0.123-0.466 |
| | Valeric acid | 0.294 | 0.029 | 0.131-0.458 |
| | LCA | 0.291 | 0.027 | 0.123-0.460 |
| | Acetic acid | 0.289 | 0.025 | 0.124-0.454 |
| | Butyric acid | 0.278 | 0.019 | 0.116-0.440 |
| | 2-Methylbutyric acid | 0.259 | 0.011 | 0.101-0.417 |
| | Isovaleric acid | 0.238 | 0.006 | 0.083-0.392 |
| | Formic acid | 0.235 | 0.005 | 0.081-0.390 |
| | Propanoic acid | 0.219 | 0.003 | 0.068-0.370 |
| | Indole-2-carboxylic acid | 0.061 | 0.000 | 0.000-0.139 |
| | 5-Hydroxyindole | 0.043 | 0.000 | 0.000-0.125 |
| | DL-5-Methoxytryptophan | 0.040 | 0.000 | 0.000-0.094 |
| | 3-(2-Hydroxyethyl)indole | 0.040 | 0.000 | 0.000-0.099 |
| AD vs. HC | Indole-3-pyruvic acid | 0.958 | 0.000 | 0.904-1.000 |
| | LCA | 0.331 | 0.047 | 0.175-0.487 |
| | 5-Hydroxytryptophan | 0.265 | 0.006 | 0.122-0.409 |
| | Valeric acid | 0.262 | 0.005 | 0.118-0.406 |
| | Butyric acid | 0.224 | 0.001 | 0.088-0.359 |
| | Isovaleric acid | 0.165 | 0.000 | 0.045-0.286 |
| | 3-(2-Hydroxyethyl)indole | 0.158 | 0.000 | 0.040-0.276 |

| | | | | |
|---|---|---|---|---|
| | 2-Methylbutyric acid | 0.155 | 0.000 | 0.041-0.268 |
| | Formic acid | 0.119 | 0.000 | 0.011-0.227 |
| | Propanoic acid | 0.111 | 0.000 | 0.015-0.206 |
| | Acetic acid | 0.105 | 0.000 | 0.013-0.198 |
| | Indole-2-carboxylic acid | 0.058 | 0.000 | 0.000-0.132 |
| | 5-Hydroxyindole | 0.049 | 0.000 | 0.000-0.128 |
| | DL-5-Methoxytryptophan | 0.036 | 0.000 | 0.000-0.083 |
| aMCI vs. AD | 3-(2-Hydroxyethyl)indole | 0.645 | 0.115 | 0.477-0.812 |
| | LCA | 0.539 | 0.672 | 0.357-0.721 |
| | 5-Hydroxyindole | 0.518 | 0.848 | 0.341-0.694 |
| | Butyric acid | 0.471 | 0.749 | 0.288-0.653 |
| | Valeric acid | 0.468 | 0.729 | 0.276-0.661 |
| | Indole-3-pyruvic acid | 0.459 | 0.654 | 0.278-0.639 |
| | 5-Hydroxytryptophan | 0.447 | 0.564 | 0.269-0.625 |
| | DL-5-Methoxytryptophan | 0.407 | 0.311 | 0.233-0.581 |
| | Indole-2-carboxylic acid | 0.395 | 0.254 | 0.220-0.571 |
| | Isovaleric acid | 0.315 | 0.044 | 0.155-0.475 |
| | Propanoic acid | 0.301 | 0.030 | 0.139-0.463 |
| | Formic acid | 0.294 | 0.025 | 0.127-0.461 |
| | 2-Methylbutyric acid | 0.287 | 0.020 | 0.132-0.442 |
| | Acetic acid | 0.247 | 0.006 | 0.093-0.401 |

Abbreviations: LCA, lithocholic acid; AUC, area under the receiver operating characteristic curve; CI, confidence interval; HC, normal cognition healthy control; aMCI, amnestic mild cognitive impairment; AD, Alzheimer’s disease.

## 3. Supplementary Figures

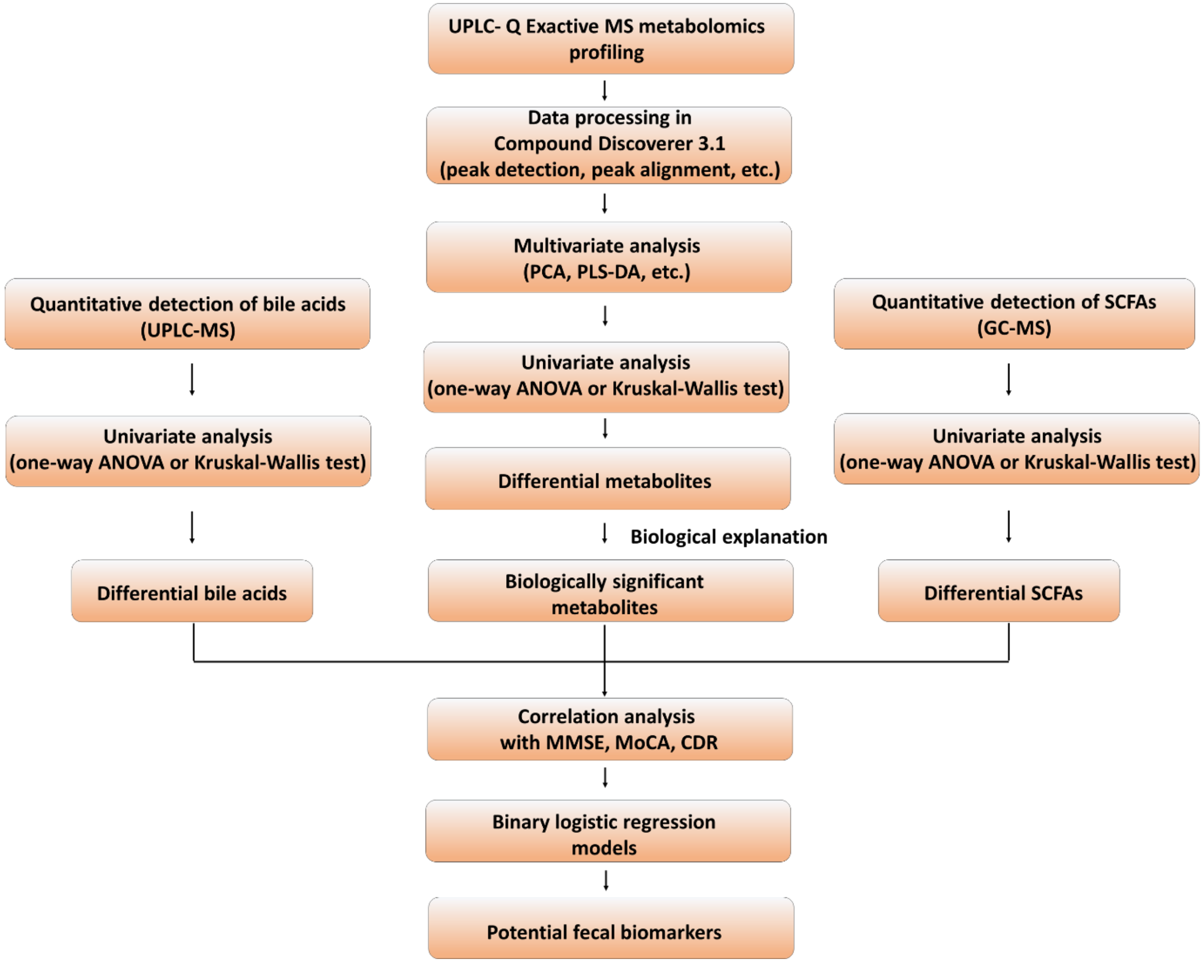

**Supplementary Figure S1. The schematic workflow of statistical analysis.** Abbreviations: UPLC, ultraperformance liquid chromatography; MS, mass spectrometry; UPLC-MS, ultraperformance liquid chromatography tandem mass spectrometry; GC-MS, gas chromatography tandem mass spectrometer; PCA, principle component analysis; PLS-DA, partial least-squares-latent structure discriminate analysis; MMSE, Mini-Mental State Examination; MoCA, Montreal Cognitive Assessment; CDR, Clinical Dementia Rating; SCFAs, short-chain fatty acids.

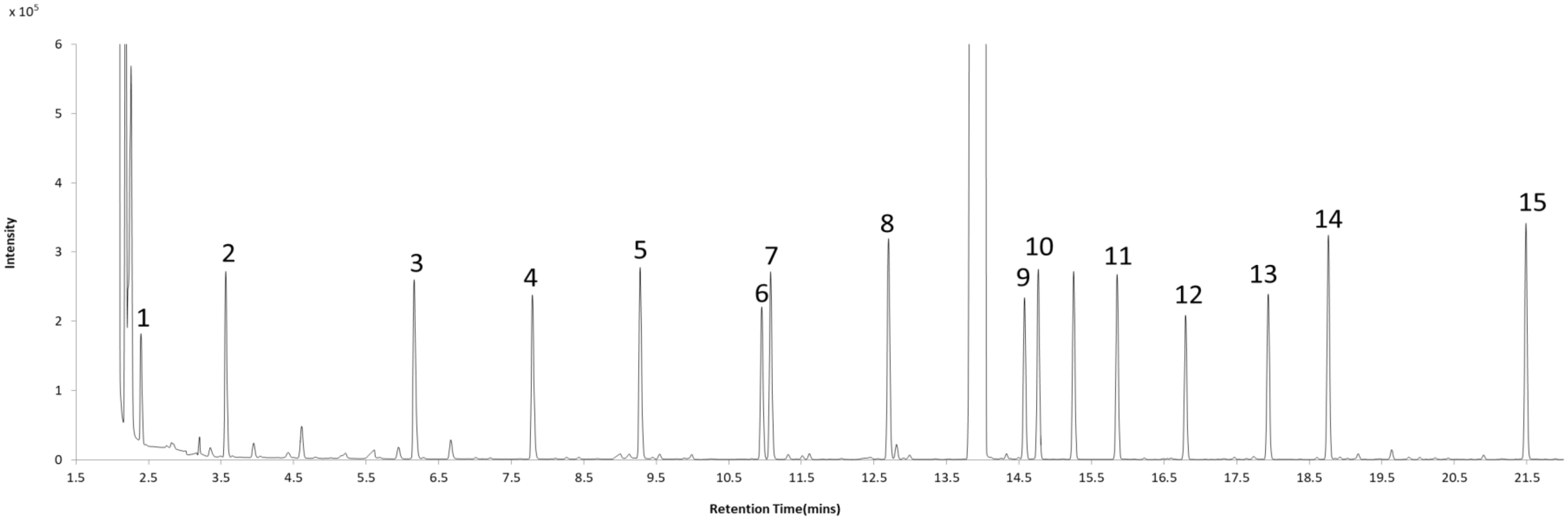


| | | | | |
|---|---|---|---|---|
| (1) Formic acid | (4) Isobutyric acid | (7) Isovaleric acid | (10) 4-Methylvaleric acid | (13) 4-Methylhexanoic acid |
| (2) Acetic acid | (5) Butyric acid | (8) Valeric acid | (11) Hexanoic acid | (14) Heptanoic acid |
| (3) Propionic acid | (6) 2-Methylbutyric acid | (9) 3-Methylvaleric acid | (12) 2-Methylhexanoic acid | (15) Octanoic acid |

**Supplementary Figure S2. The GC-MS chromatogram of fifteen SCFAs standards solution mixture.** Note: SCFAs detected in the study subjects were marked with underline. Abbreviations: GC-MS, gas chromatography tandem mass spectrometry; SCFAs, short-chain fatty acids.

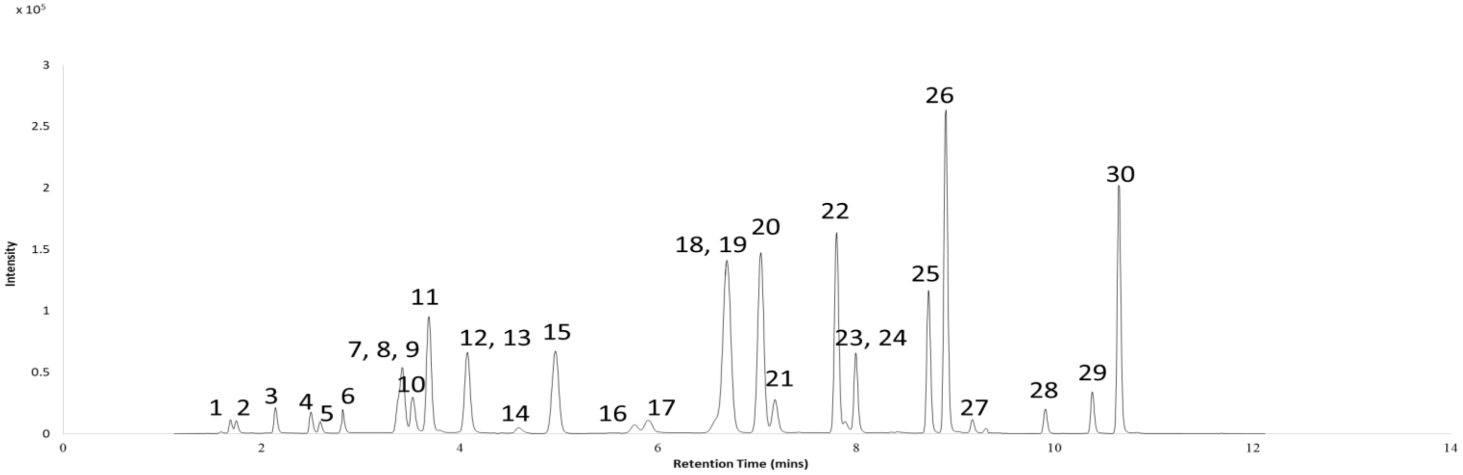


(1) Tauro-Alpha-Muricholic Acid (T-α-MCA)

(2) Tauro-Beta-Muricholic Acid (T-β-MCA)

(3) Taurohyocholic Acid (THCA)

(4) Tauroursodeoxycholic Acid (TUDCA)

(5) Taurocholic Acid (TCA)

(6) Glycohyocholic Acid (GHCA)

(7) Glycocholic Acid (GCA)

(8) Omega-Muricholic Acid (ω-MCA)

(9) Glycoursodeoxycholic Acid (GUDCA)

(10) Glycohyodeoxycholic Acid (GHDCA)

(11) Alpha-Muricholic Acid (α-MCA)

(12) Taurochenodeoxycholic Acid (TCDCA)

(13) Beta-Muricholic Acid (β-MCA)

(14) Taurodeoxycholic Acid (TDCA)

(15) Hyocholic Acid (HCA)

(16) Allocholic Acid (ACA)

(17) Cholic Acid (CA)

(18) GlycochenodeoxycholicAcid (GCDCA)

(19) Ursodeoxycholic Acid (UDCA)

(20) Hyodeoxycholic Acid (HDCA)

(21) Glycodeoxycholic Acid (GDCA)

(22) Nutriacholic Acid (nutriCA)

(23) 12-Ketodeoxycholic Acid (12-ketoDCA)

(24) Taurolithocholic Acid (TLCA)

(25) Chenodeoxycholic Acid (CDCA)

(26) Deoxycholic Acid (DCA)

(27) Glycolithocholic Acid (GLCA)

(28) Isodeoxycholic Acid (iso-DCA)

(29) Isolithocholic Acid (iso-LCA)

(30) Lithocholic Acid (LCA)

**Supplementary Figure S3. The UPLC-MS chromatogram of thirty bile acids standards solution mixture.** Note: bile acids detected in the study subjects were marked with underline. Abbreviations: UPLC-MS, ultraperformance liquid chromatography tandem mass spectrometry.

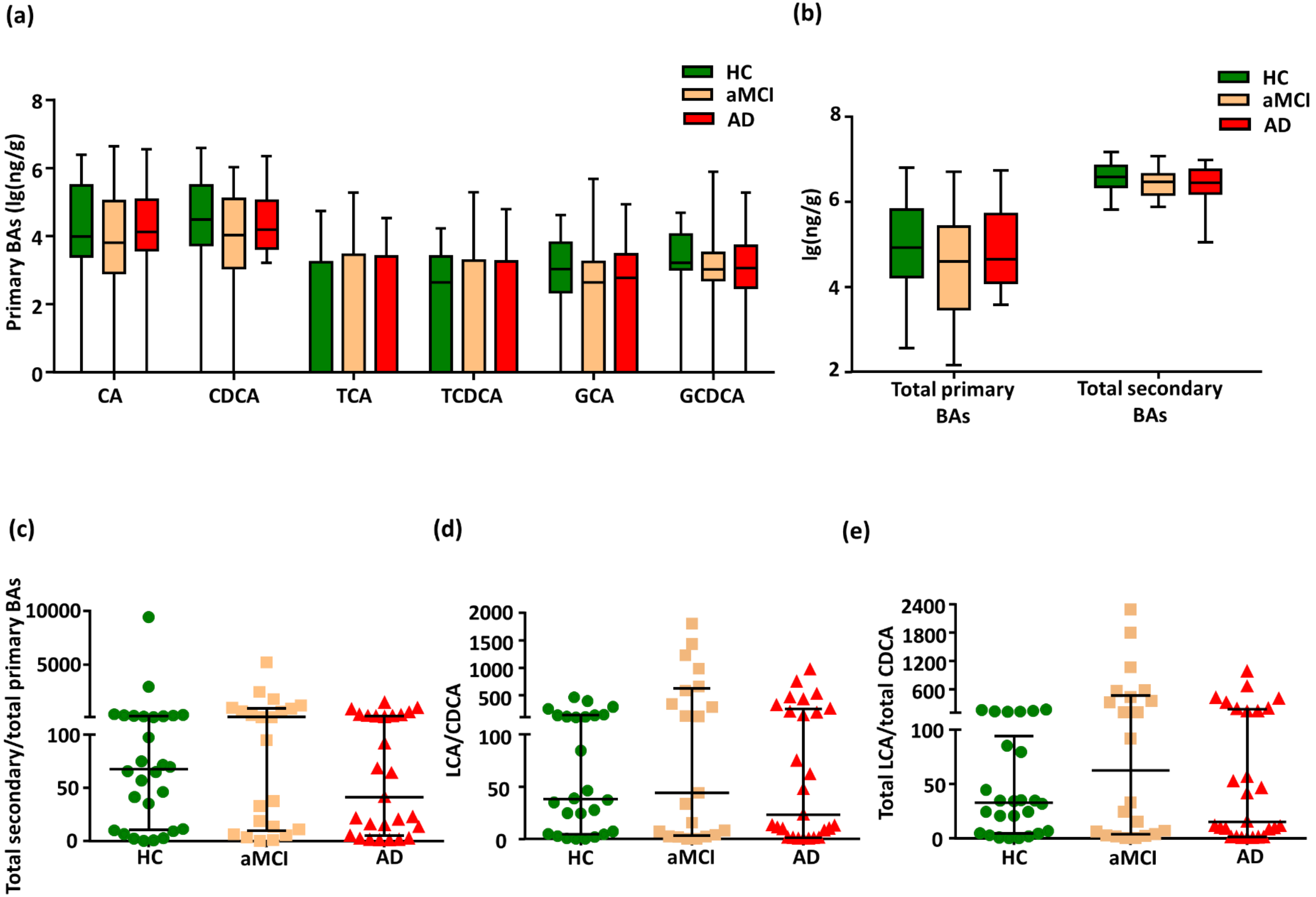
(a)
HC
aMCI
AD
Primary BAs (lg(ng/g))
8
6
4
2
0
CA
CDCA
TCA
TCDCA
GCA
GCDCA
(b)
HC
aMCI
AD
lg(ng/g)
8
6
4
2
Total primary BAs
Total secondary BAs
(c)
Total secondary/total primary BAs
10000
5000
100
50
0
HC
aMCI
AD
(d)
LCA/CDCA
2000
1500
1000
500
100
50
0
HC
aMCI
AD
(e)
Total LCA/total CDCA
2400
1800
1200
600
100
50
0
HC
aMCI
AD

**Supplementary Figure S4. Unchanged intestinal bile acids conversion in AD patients.** Comparison of fecal (a) primary bile acids, (b) calculated total primary and total secondary bile acids, (c) the ratio of total secondary to total primary bile acids, (d) the ratio of LCA to CDCA and (e) the ratio of total LCA to total CDCA among HC, aMCI and AD groups. Note: n: AD= 27, aMCI= 22, HC= 28. *P* values were determined using a one-way ANOVA or Kruskal-Wallis test. Abbreviations: BAs, bile acids; HC, normal cognition healthy control; aMCI, amnestic mild cognitive impairment; AD, Alzheimer's disease; CA, cholic acid; CDCA, chenodeoxycholic acid; TCA, taurocholic acid; TCDCA, taurochenodeoxycholic acid; GCA, glycocholic acid; GCDCA, glycochenodeoxycholic acid; LCA, lithocholic acid.

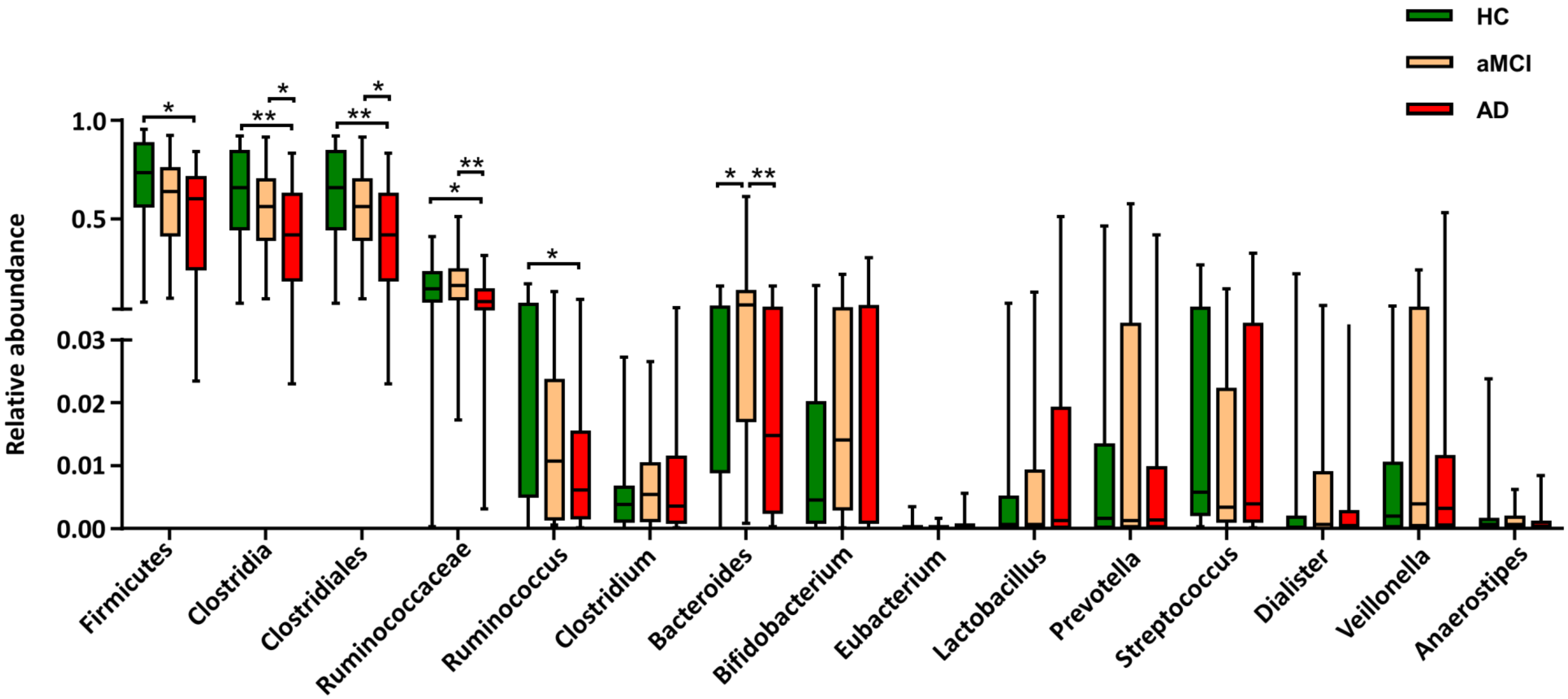
HC
aMCI
AD
Relative aboundance
1.0
0.5
0.03
0.02
0.01
0.00
*
**
*
**
*
*
**
*
*
*
**
Firmicutes
Clostridia
Clostridiales
Ruminococcaceae
Ruminococcus
Clostridium
Bacteroides
Bifidobacterium
Eubacterium
Lactobacillus
Prevotella
Streptococcus
Dialister
Veillonella
Anaerostipes

**Supplementary Figure S5. Altered SCFA-producing bacteria in AD patients.** Comparison of 15 known SCFA-producing bacteria among HC, aMCI and AD groups. Note: n: AD= 28, aMCI= 32, HC= 33. *P* values were determined using a one-way ANOVA or Kruskal-Wallis test. Abbreviations: SCFAs, short-chain fatty acids; HC, normal cognition healthy control; aMCI, amnestic mild cognitive impairment; AD, Alzheimer's disease.

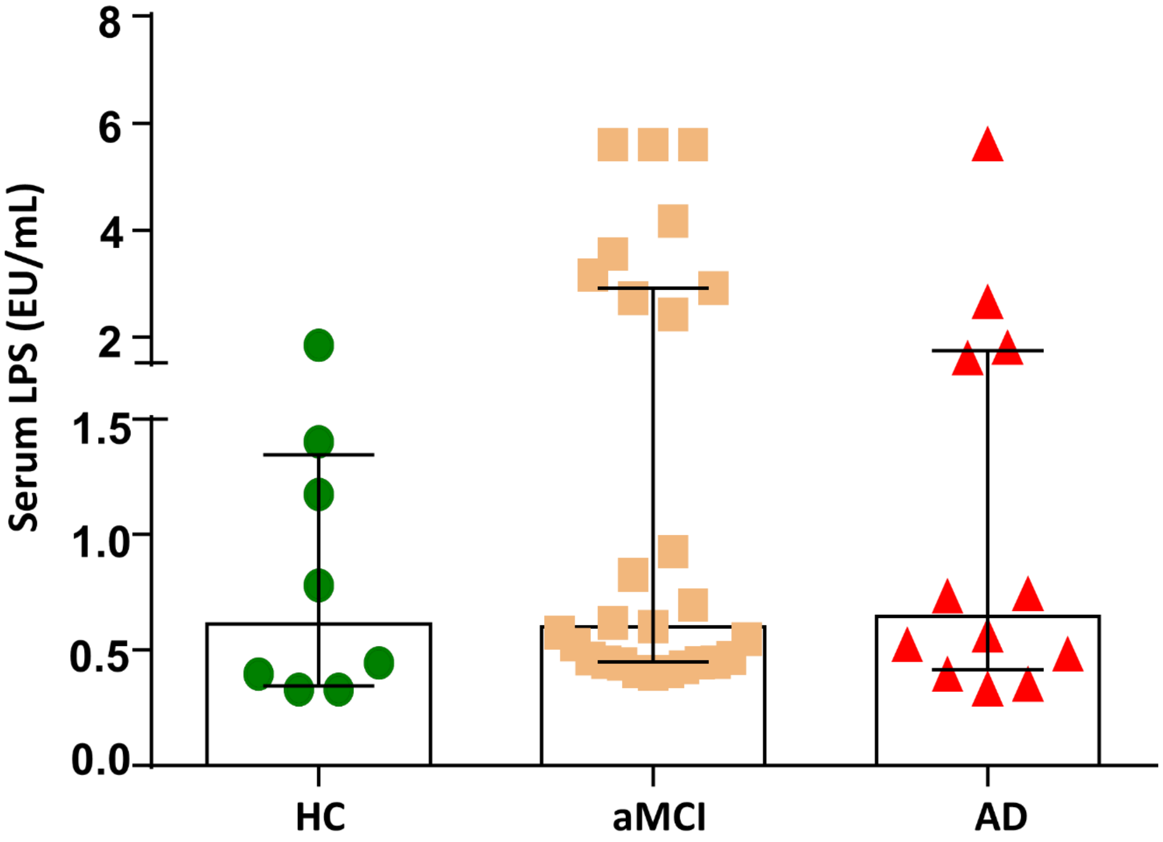
Serum LPS (EU/mL)
8
6
4
2
1.5
1.0
0.5
0.0
HC
aMCI
AD

**Supplementary Figure S6. Increased trend of circulating LPS in aMCI and AD patients.** Comparison of serum LPS level among HC (n=9), aMCI (n=27) and AD (n=12) groups. Abbreviations: HC, normal cognition healthy control; aMCI, amnestic mild cognitive impairment; AD, Alzheimer's disease; LPS, lipopolysaccharide.

## 4. Supplementary Reference


1. Association AP. Diagnostic and statistical manual of mental disorders,fourth edition(DSM-IV). Am Psych Ass 1994;42.
2. Dubois B, Feldman HH, Jacova C, et al. Research criteria for the diagnosis of Alzheimer's disease: revising the NINCDS-ADRDA criteria. The Lancet Neurology 2007;6(8):734-46.
3. Morris JC. The Clinical Dementia Rating (CDR): current version and scoring rules. Neurology 1993;43(11):2412-4.
4. Liu P, Wu L, Peng G, et al. Altered microbiomes distinguish Alzheimer's disease from amnestic mild cognitive impairment and health in a Chinese cohort. Brain, behavior, and immunity 2019;80:633-43.
5. Petersen RC. Mild cognitive impairment as a diagnostic entity. Journal of internal medicine 2004;256(3):183-94.
6. Guo N-W, Liu HC, Wong PF. Chinese version and norms of the Mini-Mental State Examination. Journal of Rehabilitation Medicine Association 1988;16:52-9.
7. Wang B, Jiang X, Cao M, et al. Altered Fecal Microbiota Correlates with Liver Biochemistry in Nonobese Patients with Non-alcoholic Fatty Liver Disease. Scientific reports 2016;6:32002.
8. M L, B W, M Z, et al. Symbiotic gut microbes modulate human metabolic phenotypes. Proceedings of the National Academy of Sciences of the United States of America 2008;105(6):2117-22.
9. Caporaso JG, Kuczynski J, Stombaugh J, et al. QIIME allows analysis of high-throughput community sequencing data. Nature Methods 2010.
10. Langille MG, Zaneveld J, Caporaso JG, et al. Predictive functional profiling of microbial communities using 16S rRNA marker gene sequences. Nature biotechnology 2013;31(9):814-21.
11. Cao H, Huang H, Xu W, et al. Fecal metabolome profiling of liver cirrhosis and hepatocellular carcinoma patients by ultra performance liquid chromatography-mass spectrometry. Analytica chimica acta 2011;691(1-2):68-75.
12. Simonato M, Fochi I, Vedovelli L, et al. Urinary metabolomics reveals kynurenine pathway perturbation in newborns with transposition of great arteries after surgical repair. Metabolomics : Official journal of the Metabolomic Society 2019;15(11):145.
13. Huang Q, Tan Y, Yin P, et al. Metabolic characterization of hepatocellular carcinoma using nontargeted tissue metabolomics. Cancer research 2013;73(16):4992-5002.
14. Furuhashi T, Sugitate K, Nakai T, Jikumaru Y, Ishihara G. Rapid profiling method for mammalian feces short chain fatty acids by GC-MS. Analytical biochemistry 2018;543:51-4.
15. Zheng X, Qiu Y, Zhong W, et al. A targeted metabolomic protocol for short-chain fatty acids and branched-chain amino acids. Metabolomics : Official journal of the Metabolomic Society 2013;9(4):818-27.
16. Lin H, An Y, Tang H, Wang Y. Alterations of Bile Acids and Gut Microbiota in Obesity Induced by High Fat Diet in Rat Model. J Agric Food Chem 2019;67(13):3624-32.
17. Penno CA, Arsenijevic D, Cunha TD, Kullakublick GA, Montani JP, Odermatt A. Quantification of multiple bile acids in uninephrectomized rats using ultra-performance liquid chromatography-tandem mass spectrometry. 2013;5(5):1155-64.
18. Zhou SY, Gillilland M, 3rd, Wu X, et al. FODMAP diet modulates visceral nociception by lipopolysaccharide-mediated intestinal inflammation and barrier dysfunction. The Journal of clinical investigation 2018;128(1):267-80.
